# A systematic evaluation of the scale invariance of texture recognition methods

**DOI:** 10.1007/s10044-014-0435-1

**Published:** 2014-12-09

**Authors:** Andreas Uhl, Georg Wimmer

**Affiliations:** Department of Computer Sciences, University of Salzburg, Jakob Haringerstrasse 2, 5020 Salzburg, Austria

**Keywords:** Scale invariance, Texture recognition, KTH-TIPS database, CUReT database

## Abstract

A large variety of well-known scale-invariant texture recognition methods is tested with respect to their scale invariance. The scale invariance of these methods is estimated by comparing the results of two test setups. In the first test setup, the images of the training and evaluation set are acquired under same scale conditions and in the second test setup, the images in the evaluation set are gathered under different scale conditions than those of the training set. For the first test setup, scale invariance is not needed, whereas for the second test setup, scale invariance is obviously crucial. The difference between the results of these two test setups indicates the scale invariance of a method (the higher the scale invariance the lower the difference). The scale invariance of the methods is additionally estimated by analyzing the similarity of the feature vectors of images and their scaled versions. Additionally to the scale invariance, we also test eventual viewpoint and illumination invariance of the methods. As texture databases for our tests we use the KTH-TIPS database and the CUReT database. Results imply that many of the considered methods are not as scale-invariant as expected.

## Introduction

Texture analysis is one of the fundamental issues in image processing and pattern recognition. Techniques for the analysis of texture in digital images are essential to a range of applications in areas as diverse as robotics, defence, medicine and geo-sciences [27].

The majority of existing texture analysis methods works with the assumption that texture images are acquired from the same viewpoint [42]. This limitation could make these methods useless for applications, where textures occur with different scales, orientations [2] or translations. Surveys about existing scale and orientation invariant texture analysis approaches are found in [32, 42]. Scale invariance is also needed in other computer vision applications like, e.g. image annotation [17, 33], object recognition [18], medical image analysis [12], et cetera.

In this work we focus on scale-invariant texture analysis approaches, even though most of the used techniques in this work exhibit additional invariance to other transformations like rotation, translation, and illumination.

**Fig. 1:**
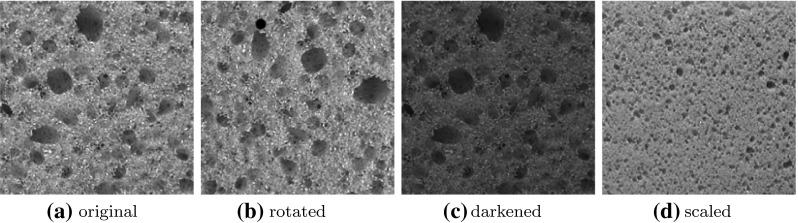
Characteristic changes by rotation (**b**), varying illumination (**c**) or by scaling (**d**) of the material bread from the KTH-TIPS database

Changing the scale of a texture has a greater impact on the characteristic of the texture than other transformations like rotating the texture or changing its illumination (see Fig. 1). This makes achieving scale invariance distinctly more challenging than achieving orientation, illumination or translation invariance.

Most of the (scale) invariant texture analysis approaches are tested on public databases like the Brodatz [4], the CUReT [5], the KTH-TIPS [11], or the UIUCTex [14] database. The scale invariance of these methods is founded on theoretical concepts, but the question is if these methods do actually exhibit scale invariance in practice. Most approaches are never really tested with respect to their effective scale invariance. If some techniques provide good results for texture databases, where textures occur at different scales, then these methods are commonly assumed to be de facto scale-invariant.

The standard setup for testing approaches on texture databases is to construct an evaluation and a training set, where the training set consists of a number of randomly chosen texture samples per texture class and the evaluation set of the remaining texture samples. If the texture database consists of texture images at different scales, and if the results of a method are good for this standard setup, does that imply that the considered approach is scale-invariant? Not necessarily. Especially, if the training set consists of a higher number of texture images per class, for nearly each image of the evaluation set there might be images of the same class in the training set with rather similar scales. This means, that a technique does not necessarily have to be scale-invariant to work well on a texture database containing textures with various scales.

Additionally, methods which are not scale-invariant may provide good results only if they are able to extract important (scale dependent) information to differentiate between textures of various classes. So, feature expressiveness might dominate the issue of scale invariance.

Several scale-invariant texture descriptors have been developed over the years. In this manuscript we assess the actual scale invariance of several approaches. We conduct experiments comparing the classification results of using identical training and evaluation sets to the results of using evaluation sets, where the images are scaled versions of the images of the training sets. In these experiments, we apply different feature extraction methods claimed to be scale-invariant to two public texture databases, the KTH-TIPS database and parts of the CUReT database.

Already in [12], well known texture recognition methods were tested with respect to their scale invariance. The focus of [12] is on the classification of celiac disease. Therefore, many of the tested texture recognition methods in [12] were adapted versions of the original proposed methods, optimized for the classification of celiac disease using celiac disease databases. Also in [23] methods are tested with respect to their scale invariance (and rotation and viewpoint invariance), but only descriptors for local interest regions are considered and the focus is not on texture recognition.

In this paper we focus on general texture recognition and will analyze the scale invariance of the original proposed methods using well known public texture databases. Contrary to [12] and [23], we will conduct additional experiments to detect the reasons for the scale invariance or missing scale invariance of the employed methods. In these experiments we use some novel image feature invariance metrics to detect the weaknesses and the strengths of the methods. These novel invariance metrics analyze the effect of changing the scale conditions of images to the outputs of the methods combined with

- constant viewpoint and illumination conditions,
- changing viewpoint and constant illumination conditions,
- changing illumination and constant viewpoint conditions,
- and changing viewpoint and illumination conditions.

A welcome byproduct of these tests is that we are able to assess the viewpoint and illumination invariance of the methods.

Most of the employed features can be used for many applications. So our results with respect to the scale, viewpoint and illumination invariance of the features could be helpful in many practical applications of the employed features like, e.g. face and facial expression recognition [16, 31], object recognition [3], medical image analysis [12], et cetera.

The contributions of this manuscript are as follows:

- We conduct tests to evaluate the scale invariance of methods dispersed in literature in a uniform and fair setting. Most of these methods were never tested with respect to their scale invariance, although it was claimed that they are scale-invariant. Actually, we reveal that the claimed scale invariance of most of the methods cannot be verified in our tests.
- We present a short and clear description of the employed methods and discuss reasons why some of the methods are not as scale-invariant as they should be according to their theoretical concept for scale invariance. Most of these reasons were not mentioned in the publications of the methods.
- We propose some novel image feature invariance metrics especially designed to detect the weaknesses and the strengths of the methods, especially the scale-, viewpoint- and illumination invariance of the methods. We show that most of the methods have big problems with changing illumination conditions, whereas changing viewpoint conditions seem to be quite unproblematic for most of the methods.

This paper is organized as follows: Sect. 2 provides a theoretical analysis of scale invariance in image processing. In Sect. 3 we briefly review a significant amount of scale-invariant texture descriptors as proposed in literature. The experimental setup, the used databases and the results are presented in Sect. 4. Section 5 presents the discussion and Sect. 6 concludes our work.

## Theoretical analysis of scale invariance

Scale invariance in image processing means that the description of objects or textures shown in images does not change if the distance between the objects or textures and the camera (or the zoom of the camera) is multiplied by a factor $f$. In this article we will focus on the scale invariance with respect to textures and ignore objects.

**Fig. 2:**
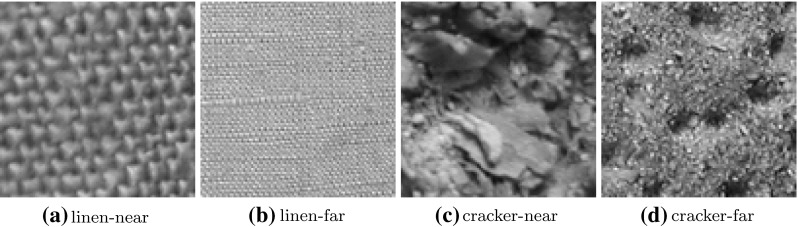
Images of the materials linen and cracker from the KTH-TIPS database with a scale difference of factor $f=4$

Of course, absolute scale invariance is practically impossible, since the resolution of an image is limited and the surface of objects and textures changes too significantly in case of higher scale factors [26]. For example the area of a texture shown in a $256 \, \times \, 256$ image is in an image showing the same texture but with a 128 times higher distance of the camera to the texture only of size $2 \, \times \, 2$. Of course it is impossible to recognize that these two images with their huge scale differences to each other show the same kind of texture. Furthermore, we perceive objects and textures in the world as meaningful only over certain ranges of scale [15]. A simple example is the bark of a tree. It is meaningless to discuss the bark at the nanometer or kilometer level. At those scales it is more relevant to talk about the molecules that form the bark or the forest in which the tree with the considered bark grows. Also smaller differences in scale have a big impact on the characteristic of textures, as for example can be seen in Fig. 2, where a scale factor of only * f* = 4 is used.

As already mentioned before, in this article we want to analyze the scale invariance of several well known texture descriptors. Scale invariance for texture descriptors is a quite demanding feature and can be achieved only for moderate scale changes.

We define a texture descriptor as being scale-invariant, if the distances between the feature vectors of images from a single texture class compared to the distances between feature vectors of different texture classes are not influenced by the fact whether the images are all gathered under one or under different scale conditions.

## Scale-invariant texture descriptors

### Scale-invariant wavelet-based methods

**Fig. 3:**
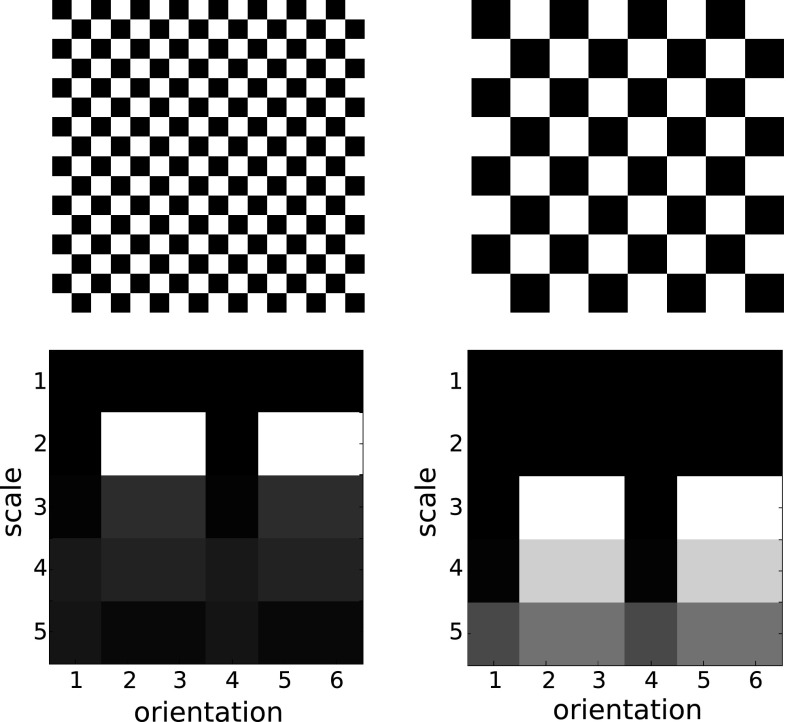
Cyclic shifting of the means of the subbands across the scale dimension

In this section we describe scale-invariant texture descriptors, that are based on multi-scale and multi-orientation wavelet transforms like the discrete wavelet transform and the Gabor wavelet transform. The subbands, resulting from these transforms, contain information at different scales and orientations of an image. The strategies to make these transforms invariant to scale changes are to reorder the corresponding transform coefficients or to find a different representation for the image before applying the respective transform. The underlying principles of achieving scale invariance are similar for the approaches in this section (except for the approach that re-arranges the image before the wavelet transform). If an image is scaled, then the subbands of the scaled image are shifted across scale dimension compared to the subbands of the unscaled image. In the first row of Fig. 3 we see two checkerboard patterns, where the right pattern is a scaled version of the left one with a scale factor of two. The second row shows the corresponding subband means of a Gabor wavelet transform. We can see that the subband means of the scaled checkerboard pattern (the right one) are shifted one scale level up compared to the subband means of the unscaled checkerboard pattern (the left one).

#### The dominant scale approach

Let the total energy of scale level (=decomposition level) $l$ be defined as the sum of the energies of the subbands of the Steerable Pyramid Decomposition [8] with scale level $l$. Then the dominant scale is the scale level with the highest total energy [24]. Means and standard deviations of the subbands are used as features. Dealing with the assumption that the subband features are periodic across the scale dimension, scale invariance is proposed to be achieved by aligning the feature elements according to the dominant scale of the input texture [24].

However, we conducted experiments using the CUReT and the KTH-TIPS database which showed that scale level 1 is nearly always the dominant scale. That of course makes this approach nearly senseless, since a new feature alignment hardly ever occurs.

#### The slide matching approach

**Fig. 4:**
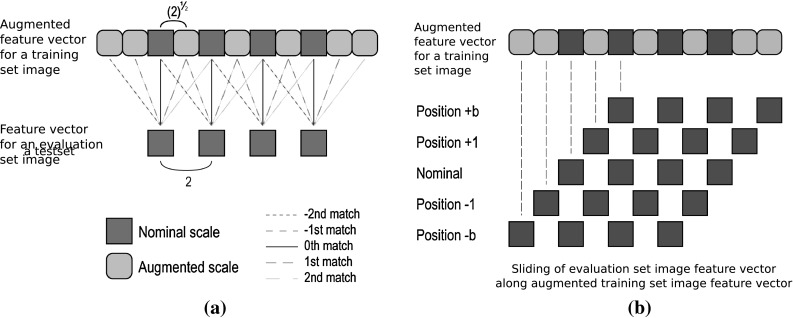
**a** Different scaling factors are used for the training set images and for the evaluation set images. Each node denotes two elements, a sum of means and a sum of standard deviations. ** b** The sliding of evaluation set image feature vector along augmented training set image vector

The approach, presented in [9], is first made orientation invariant by summing up the means and standard deviations of the subbands of the Gabor Transformation [7] with same scale level. Images of the evaluation and the training set are filtered using two different Gabor kernels. The difference between the two Gabor kernels is, that the training set kernel has additional scale levels in between the scale levels of the evaluation set kernel (see Fig. 4a). The distance between an image of the training set and an image of the evaluation set is the distance that is minimized by sliding the feature vectors along the scale dimension against each other (see Fig. 4b).

However, we conducted experiments which showed that there is only a small tendency that the feature vectors are slided in the correct direction along the scale dimension to balance scale-scale differences between two images.

#### The log-polar approach

The log-polar transformation maps point in the Cartesian plane to points in the log-polar plane. The new (log-polar) coordinate system has the properties in that scaling and rotations (in the Cartesian plane) are converted to translations.

Now scale invariance (and orientation invariance) can be achieved by analyzing the transformed image with a row shift invariant method, the adaptive row shift invariant wavelet packet transform [28]. This transform is similar to the wavelet packet transform, but it additionally applies the wavelet decomposition to a row shifted version of each subband. The row shift invariant wavelet packet transform is combined with the best basis algorithm. The feature vector of an image consists of the subband energies.

### Scale-invariant methods based on fractal analysis

**Fig. 5:**
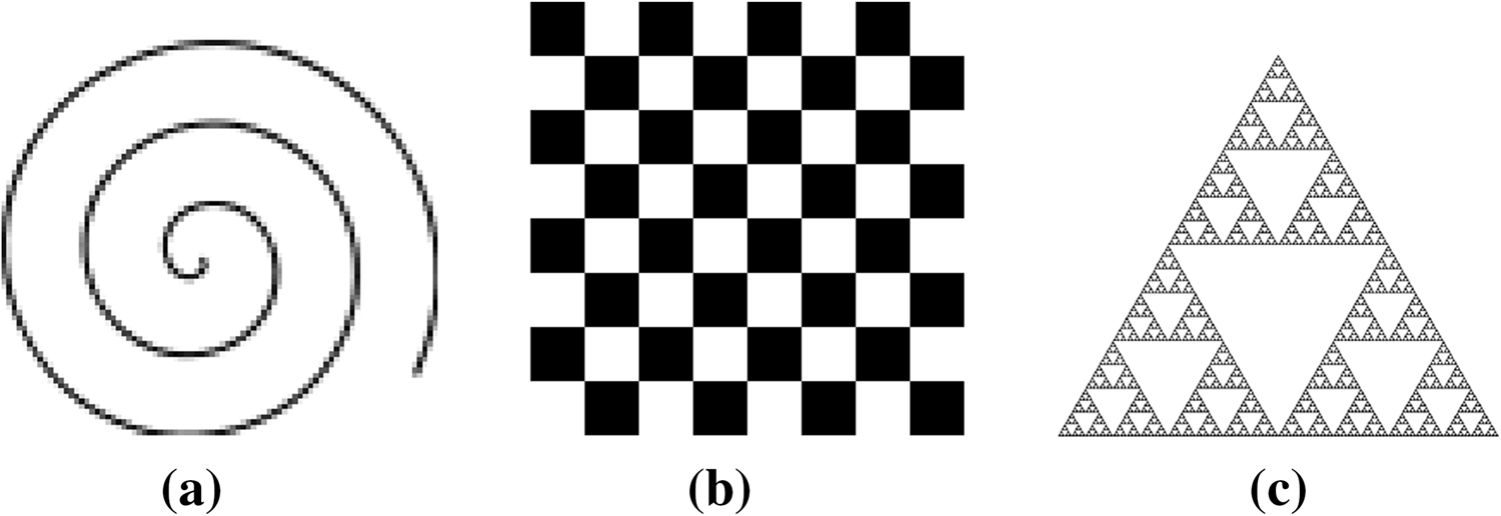
Fractal dimension $D$ in 2D space. ** a** Smooth spiral curve with $D \, = \, 1$, ** b** the checkerboard with $D \, = \, 2$ and **c** the Sierpinski-Triangle with $D \, \approx \, 1.6$

For a point set * E* defined on $\mathbb {R}^2$, the fractal dimension of $E$ is defined as:

1 $$\begin{aligned} dim(E)=\lim _{\delta \rightarrow 0} \frac{\log N(\delta ,E)}{-\log \delta }, \end{aligned}$$

where $N(\delta ,E)$ is the smallest number of sets with diameter less than $\delta$ that cover $E$. The set is made up of closed disks of radius $\delta$ or squares of side length $\delta$. In Fig. 5 we see some examples for the fractal dimension of different objects.

Two of the three methods presented in this section are based on a special fractal feature, the local fractal dimension, also called the local density function. Let $\mu$ be a finite Borel regular measure on $\mathbb {R}^2$. For $x\in \mathbb {R}^2$, denote $B(x,r)$ as the closed disk with center $x$ and radius $r>0$. $\mu (B(x,r))$ is considered to be an exponential function of $r$, i.e. $\mu (B(x,r)) \, = \, c\, r^{D(x)}$, where $D(x)$ is the density function and $c$ is some constant. The local density function of $x$ is defined as

2 $$\begin{aligned} D(x) \, = \, \lim _{r\rightarrow 0} \frac{\log \mu (B(x,r))}{\log r}. \end{aligned}$$

The density function measures the “non-uniformness” of the intensity distribution in the region neighboring the considered point.

The local density $D$ (or also called the local fractal dimension) is invariant under the bi-Lipschitz map, which includes view-point changes and non-rigid deformations of a texture surface as well as local affine illumination changes. Consequently, the local fractal dimension is especially interesting for developing scale-invariant feature descriptors. However, the proof for invariance under the bi-Lipschitz map in [39] shows only that the local fractal dimension is invariant in a continuous scenario, but not in case of a discrete scenario (e.g. an image).

#### The multi-fractal spectrum

Three different types of measures $\mu (B(x,r))$, each extracting different kinds of information, are used for the computation of the local density. These measures are:

3 $$\begin{aligned} \mu _{1}(B(x,r))& \, = \, \int _{B(x,r)} I(\sigma ) \,dx \end{aligned}$$

4 $$\begin{aligned} \mu _{2}(B(x,r))& \, = \, \int _{B(x,r)} \sum \nolimits _{k \,= \, 1}^{4}(f_{k} \, \times \, (I(\sigma )^{2})^{\frac{1}{2}} \,dx \end{aligned}$$

5 $$\begin{aligned} \mu _{3}(B(x,r))& \, = \, \int _{B(x,r)}\left| \left( I_{xx}(\sigma ) \, + \, I_{yy}(\sigma ) \right) \right| \,dx, \end{aligned}$$

where $I(\sigma )$ is the Gaussian blurred image $I$ using variance $\sigma ^2$, $I_{xx}(\sigma )$ and $I_{xx}(\sigma )$ are the second derivatives in $x$-direction respectively $y$-direction, ‘×’ is the 2D convolution operator and $\{f_{k},\,k \, = \, 1,2,3,4\}$ are four directional operators (derivatives) along the vertical, horizontal, diagonal, and anti-diagonal directions.

Let $E_{\alpha }$ be the set of all image points $x$ with local density in the interval $\alpha$:

$$\begin{aligned} E_{\alpha } \, = \, \{x\in \mathbb {R}^{2}:\, D(x)\in \alpha \}. \end{aligned}$$

Usually this set is irregular and has a fractional dimension $f(\alpha )=dim(E_{\alpha })$.

The feature vector of an image $I$ consists of the concatenation of the fractal dimensions $f(\alpha _{i})$ for the three different measures $\mu (B(x,r))$ [39].

#### Fractal analysis using filter banks

First the images are filtered with the MR8 filter bank [10, 35], a rotationally invariant, nonlinear filterbank with 38 filters but only 8 filter responses [34]. However, filtering has the drawback of lowering the level of bi-Lipschitz invariance.

Then the local densities are computed. So for each pixel of an image there is an 8-dimensional local density vector. For each class of the training set, the local density vectors of the images belonging to a class are aggregated and then cluster centers (called textons) are learned by k-means clustering. Given an image, its corresponding model (i.e. the histogram) is generated by labeling each of its local density vectors with the texton that lies closest to it [34].

Distances between two frequency histograms (models) are measured using the $\it \chi ^{2}$ statistic.

#### Fractal dimensions for orientation histograms

**Fig. 6:**
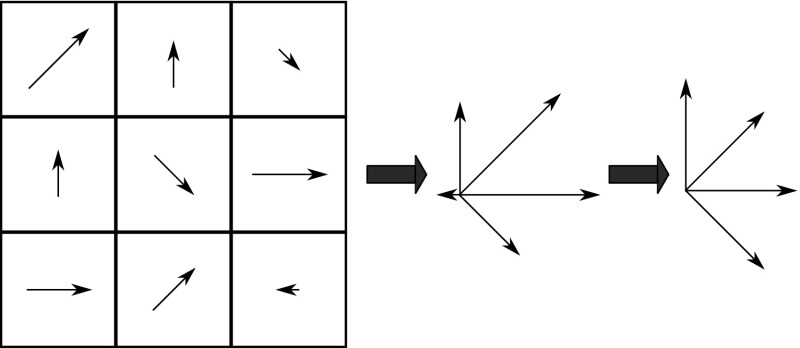
The process of constructing and discretizing the orientation histogram when using the neighborhood of size $3 \, \times \, 3$

Similar to SIFT features (see Sect. 3.3.1), this method [38] is based on first computing local orientation histograms. An orientation histogram from the neighborhood of a given pixel is formed by discretizing orientations by weighing the gradient magnitude (see Fig. 6).

The histogram is then assigned to one of 29 orientation histogram templates, which are constructed based on the spatial structure of the orientation histogram.

29 binary images are generated by setting a pixel to one if it is assigned to template $i$ ($i \in \{1,\ldots ,29\}$) and to zero otherwise. Then the fractal dimensions of the binary images is computed by means of the box counting algorithm^1^. This process is applied for eight different neighborhood sizes (scale levels). In order to get better robustness to scale changes, finally a wavelet transform (a redundant tight wavelet frame system) is applied across the scale dimension (the different neighborhood sizes) of the fractal dimensions.

The final feature vector of an image consists of the approximation and detail coefficients of the wavelet transform.

However, it is questionable if the wavelet transform really increases the scale invariance. We conducted experiments using the CUReT database that showed that there is no difference between the classification results of the method with or without the wavelet transform.

### Other approaches

In this section we present approaches that are neither based on wavelet transforms nor on fractal analysis. The first two approaches are based on the widely used SIFT features [18] and affine invariant region detectors [41], two approaches work with neural networks [19, 40] and one approach analyzes characteristics of connected regions (blobs) [37].

#### SIFT features

The Scale Invariant Feature Transform (SIFT) [18] is probably the most popular feature used in computer vision [36]. SIFT detects salient image regions (key points) and extracts discriminative yet compact descriptors of their appearance. SIFT key points are invariant to viewpoint changes like translation, rotation, and rescaling of an image.

By means of detecting the maxima/minima of the Difference of Gaussians (DoG), local scale space extrema are found. Then orientation histograms are formed on the basis of the neighboring regions of detected key points. The original approach [18] is suited for object recognition, but not for texture recognition.

In particular, the keypoint/region detection of SIFT is not appropriate for texture images. We test two different ways to deal with that problem:

1. We use dense SIFT features [6], which means that SIFT descriptors are computed for each pixel of an image.
2. We use a region detector that is suited for texture images and then the SIFT descriptor is applied to the detected regions [41].

A region detector suited for texture recognition is the Harris detector [14, 21, 41]. The Harris detector is based on the second moment matrix $M$. The Laplacian scale selection finds the characteristic scale at the interesting points by maximizing the Laplacian-of-Gaussian. The elliptic region around a location found is described by its principal axes corresponding to the eigenvectors of $M$ and axis length depending on the eigenvalues. For affine invariance, a region is normalized by mapping it onto a unit circle and using the SIFT descriptor to describe the region. It should be noted that instead of using the Harris detector it would be possible to use other region detectors (e.g. Laplacian [41] and Hessian region detectors [22]) and descriptors (e.g. SPIN and RIFT features [41]).

For both ways, using dense SIFT features or using the Harris detector, SIFT descriptors are computed. Both approaches follow the strategy applied in Sect. 3.2.2 (building a texton dictionary by clustering the SIFT descriptors, followed by generating models for each image). In case of the Harris detector, this strategy is one of the used strategies in [41], in case of the dense SIFT features, this strategy is different to the classical dense SIFT approach [6]. However, properties with respect to scale invariance should not be changed.

We denote the approach using the dense SIFT features as “Dense SIFT Features” and the approach using the Harris detector as “Local Affine Regions”.

#### Pulse-coupled neural networks based methods

Pulse-coupled neural networks (PCNN’s) [29] are neural models inspired by the visual cortex of a cat. PCNN is a neural network algorithm that produces a series of binary pulse images when stimulated with an image. The intersecting cortical model (ICM) [19] and the spiking cortical model (SCM) [40] are two methods derived from the PCNN, that are faster and provide higher or similar results as compared to the PCNN (see [19] and [40]).

The ICM and SCM models consist of two coupled oscillators, a small number of connections and a non-linear function. The final feature vectors of the SCM and ICM consist of the entropies of the binary output images.

The authors in [19] and [40] state that their approaches (ICM and SCM) are scale-invariant (and rotation and translation invariant); however, their manuscripts miss a justification for this statement. They cited a further publication [13], in which scale invariance is explained. The problem is that in this publication a special kind of PCNN is considered and that scale invariance is only shown for objects on a uniform background, not for textures.

#### Multiscale blob features

In order to derive multiscale blob features [37], a series of flexible threshold planes are applied to a textured image and then the topological and geometrical attributes of the blobs in the obtained binary images are used to describe image texture.

Flexible threshold planes $I_{\rm FP}(\sigma )$ are determined by Gaussian blurring an image $I$ using different variances $\sigma ^{2}$ followed by adding biases $b$. By applying the flexible threshold planes to the gray scale image $I$, binary images are obtained.

$$\begin{aligned} g_{b}(x,y;\sigma ) \, = \, {\left\{ \begin{array}{lll} 1\,\hbox { if } I(x,y) > I_{\rm FP}(x,y;\sigma ,b)\\ 0\,\hbox { otherwise } \end{array}\right. } \end{aligned}$$

**Fig. 7:**
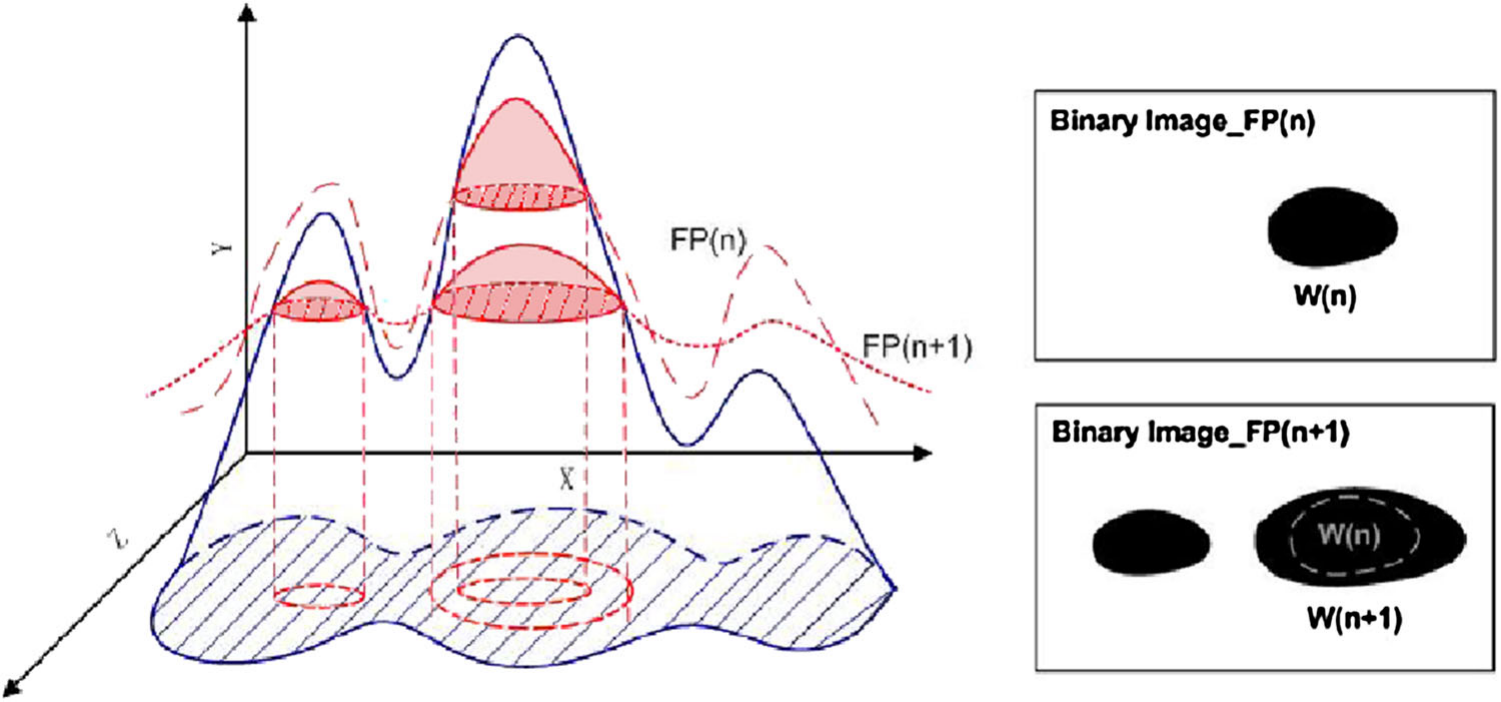
Generating binary blob images

In each binary image all 1-valued pixels and 0-valued pixels are grouped into two sets of connected regions called blobs (see Fig. 7). Two features are used to describe an image, the number of blobs and the shapes of the blobs. The shape features are invariant to spatial scaling within a small range, but the number of blobs changes to some extent. The final feature vector used in [37] is the combination of the number and shape of the blob, but we also carry out experiments using just the scale-invariant shape features as stand-alone features.

### Scale-dependent methods

To estimate the extent of scale invariance of the claimed scale-invariant methods, we additionally evaluate the scale invariance of some scale-dependent methods as reference values. We decided to evaluate three commonly used methods to describe textures: Local Binary Pattern (LBP) [25] (the standard LBP approach with block size = 3), the Gabor Wavelet Transform [7] (3 scale levels, means and standard deviations are used as features of the subbands) and the Dual-Tree Complex Wavelet Transform (DT-CWT) [30] (6 scale levels, means and standard deviations are used as features of the subbands).

## Experimental analysis

### Experimental setup

We use the software of the Robotic Research Group^2^ for region detection (Harris detector) and description (SIFT) in Sect. 3.3.1, the VLFEAT implementation [36] for the dense SIFT features in Sect. 3.3.1, and the implementation of Geusebroek et al. [10] for the MR8 filter in Sect. 3.2.2. The remaining algorithms are implemented specifically for this work following the descriptions in the respective publications (using Matlab).

For a better comparability of the results, all methods are evaluated using a kNN-classifier (original manuscripts employ a wide variety of different classifiers of course). Classification accuracy is computed using an evaluation set and a training set. An image from the evaluation set is classified into the class, to which most of the $k$ nearest neighbors from the training set belong. To balance the problem of varying results depending on $k$, we average the 10 results of the kNN-classifier using *k* = 1–10.

For the algorithms using * k*-means clustering, the results are changing each time they are applied. For these methods we provide average results from 10 runs per method.

As already mentioned before, it is not adequate to state that an approach is scale-invariant just because the results are good for databases containing images with various scales. These databases usually contain a high number of image samples per texture class (e.g. 81 samples for the KTH-TIPS database and 40 samples for the UIUCTex database). Even after dividing each class into one part for the training set and one part for the evaluation set, it is most likely that for an image of the evaluation set there will be at least one image of the training set, which has a similar scale and belongs to the same class. This means, that a technique does not necessarily have to be scale-invariant to work well on databases, which contain images of various scales.

For explicitly testing scale invariance of an approach, we need to use databases which provide the information about the scale an image belongs to. With this information we are able to divide these databases into one part for the training set and one part for the evaluation set, where the training set contains differently scaled images than the evaluation set. This is the reason for choosing the Columbia-Utrecht (CUReT) database [5] and the KTH-TIPS database [11] for our tests.

Another possibility to construct a training and an evaluation set containing differently scaled images would be to synthetically scale an arbitrary texture database, but this changes the characteristics of the images too much (e.g. interpolation effects, eventual contrast changes, etc. ...).

### The CUReT database

The Columbia-Utrecht (CUReT) database contains images of 61 materials and includes many surfaces. The CUReT database has a ditional scaled data (scale factlarge variety of textures. Each texture is imaged under 205 different viewing and illumination conditions, but without significant scale changes. We consider a subset of the CUReT database, called the cropped CUReT database^3^, with only 92 viewpoint and illumination conditions per texture, where the azimuthal viewing angle is less than 60°. A central $200 \, \times \, 200$ region is cutted out for each of the selected images and is used instead of the original image.

For four texture classes of the CUReT database (material numbers 2, 11, 12, and 14), additional scaled data (scale factor $f \, \approx \, 1.75$) is available (material numbers 29, 30, 31, 32).

**Fig. 8:**
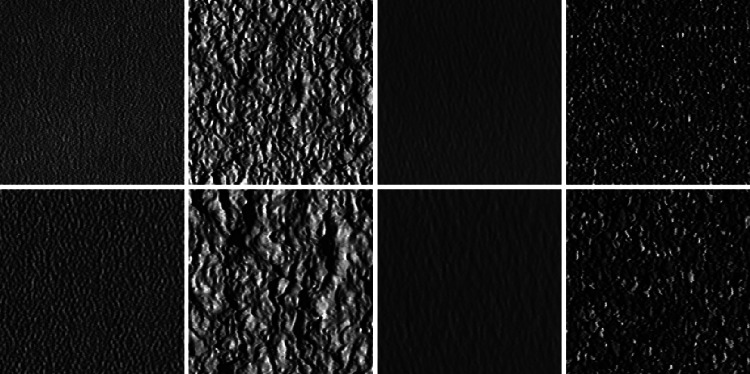
The* top row* shows one texture image per material (as material numbers 2, 11, 12, and 14) from the CUReT database (originally scaled), while the* bottom row* shows these textures with a higher zoom factor (as material numbers 29, 30, 31, and 32)

With these materials we want to test the scale invariance of the reviewed approaches. The materials are shown in Fig. 8.

For explicitly testing scale invariance, we use the original textures ($4 \, \times \, 92$ images, images of material numbers 2, 11, 12, and 14) as training set. As evaluation set we either also use the original textures (test setup 1) or we use the scaled versions of the original textures (test setup 2).

For each image of the training set, the according image of the evaluation set from the same material (no matter if original or scaled) with the same viewing and illumination condition is not accepted as nearest neighbor for the kNN-classifier. That means we have a leave-one-out cross-validation (LOOCV) in case of test setup 1 (evaluation set and training set are identical and it is not allowed that an image is the nearest neighbor of itself) and in test setup 2 the scaled version of an image is not allowed as nearest neighbor for the original version of that image.

For test setup 1 scale invariance is not needed, while for test setup 2 scale invariance is obviously crucial, since the evaluation set consists of differently scaled data than the data in the training set. A small difference between the classification results of test setup 1 and 2 indicates high scale invariance and a high difference indicates low scale invariance.

In Table 1 we see the overall classification rates (OCR) for our Experiment. Since the reviewed methods have been developed and optimized for different databases, the accuracies using the two different evaluation sets are not very relevant, but the (relative) differences between the accuracies of the two evaluation sets, indicating the extent of scale invariance, are very interesting. The relative differences in Table 1 (‘Diff.’) are computed as follows:

$$\begin{aligned} d \, = \, 100 \, \frac{r_{\rm setup \, 1} - r_{\rm setup \,2}}{r_{\rm setup \,1}}, \end{aligned}$$

where $d$ denotes the relative difference and $r_{\rm setup}$ the result of a method using the according test setup.

Additionally, we want to assess statistical significance of our results. The aim is to analyze if the images of setup 1 are classified differently to these of setup 2 by the various methods considered (high effect of scale differences to the classification results of the images), or if the images are classified similarly, despite of the differences in scale (low effect of scale differences to the classification results of the images). We use the McNemar test [20] to evaluate if a method classifies the images of setup 1 significantly different to those of setup 2 for a given level of significance ($\alpha \, = \, 0.01$) by building test statistics from incorrectly classified images. The outcome of an image used for the McNemar test is the most frequently occurring outcome of the 10 kNN-classification results for the considered image using* k* = 1–10.

The results shown in Table 1 are quite unexpected. Many methods designed to be scale-invariant, turn out to be less scale-invariant than the three scale-dependent methods. Especially the DT-CWT technique provides more scale invariance in the experiment than any of the methods designed to be scale-invariant. Additionally, the highest OCR is achieved. Also the Gabor Wavelets provide more scale invariance than most of the methods designed to be scale-invariant.

**Table 1:** OCR results for the two experiments on the CUReT database. The (relative) differences between the results indicate the scale invariance of the methods

Method	Setup 1	Setup 2	Diff.
Dominant scale approach	97.0	89.9	7.3
Slide matching	99.2	81.1	18.3
Log-polar approach	82.3	75.4	8.4
Multi-fractal spectrum	100	87.7	12.3
Fractal analysis using filter banks	99.9	73.9	26.0
Fractal dim. for O. histograms	97.6	71.8	26.4
Dense SIFT features	93.7	59.8	36.2
ICM	97.1	72.2	25.6
SCM	100	94.2	5.8
Multiscale blob feat. (shape and* n*.)	100	86.8	13.2
Multiscale blob feat. (shape)	99.7	93.5	6.2
Local affine regions	98.2	88.7	9.7
Local binary pattern	99.9	79.1	20.8
Gabor wavelet	99.8	89.5	10.3
DT-CWT	100	97.9	2.1

The scale-dependent methods based on wavelet transforms seem to be more scale-invariant than the wavelet transform-based methods, which are explicitly designed to improve scale invariance. At least two of the three methods using fractal analysis are not rated to be scale-invariant, in contrast to their theoretical concept. Dense SIFT Features provide hardly any scale invariance in our experiment, while the keypoint-based variant exhibits at least scale invariance to some extent. Multiscale Blob Features are more scale-invariant when using only the shapes of the blobs as features than using the shape and number of the blobs found, which corresponds to the theoretical considerations. The SCM is distinctly more scale-invariant than the ICM approach.

The McNemar test showed that the only method without a significant difference between the outcomes of the images from setup 1 and setup 2 is the DT-CWT method.

### The KTH-TIPS database

**Fig. 9:**
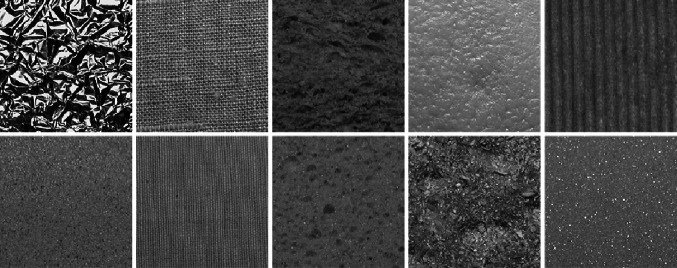
The 10 materials of the KTH-TIPS database

The KTH-TIPS database [11] contains images of 10 materials (see Fig. 9).

**Fig. 10:**
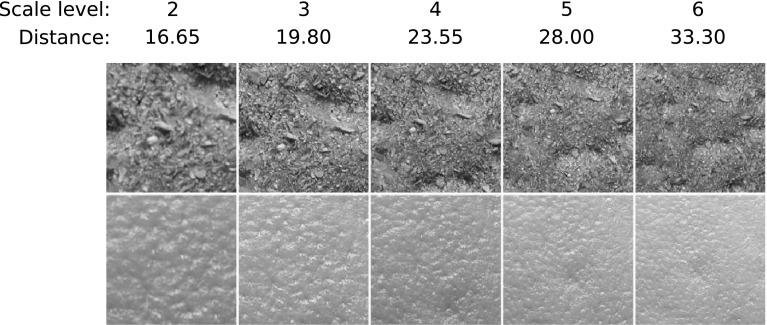
Scale levels 2–6 (from* left* to* right*) for the materials cracker and orange peel of the KTH-TIPS database

It provides variations in scale as well as variations in pose and illumination. Images were taken at 9 different scales spanning two octaves (see Fig. 10). At each scale level 9 images were taken in a combination of three poses (frontal, rotated 22.5° left and rotated 22.5° right) and three illumination conditions (from the front, from the side at roughly 45° and from the top at roughly 45°). Similar to the experiments with the CUReT database, we only consider a cropped version of the database^4^. A central $200\times 200$ region is cutted out for each of the images and is used instead of the original image.

We only use the images with scale levels 2–6 (since images of scale level 1 are often blurred and the images of scale levels 7–9 are much smaller than $200 \, \times \, 200$ pixels in case of some materials). Two examples of the scale levels 2–6 are shown in Fig. 10.

For explicitly testing scale invariance, we conduct experiments similarly to these for the CUReT database. We divide the KTH-TIPS database into 5 sub-databases $\rm SD_{i},\, i\in \{2,\ldots ,6 \}$, where sub-database $\rm SD_{i}$ consists of the texture images with scale level $i$. As training set we use sub-database $\rm SD_{6}$ and as evaluation sets one of the 5 sub-databases $\rm SD_{i}$. For each image of the training set, the according image of the evaluation set from the same material with same pose and illumination is not accepted as nearest neighbor for the kNN-classifier (for example, if $\rm SD_{6}$ is used as evaluation set, we perform a LOOCV).

In Table 2 we see the results of that approach. The five columns of ‘evaluation sets’ show the classification results when using the subsets $\rm SD_{i}\, i\in \{2,\ldots ,6\}$ as evaluation sets and $\rm SD_{6}$ as training set. The columns $\rm SD_{i}\, i\in \{2,\ldots ,5\}$ of ‘decrease by scaling’ show how much the results decrease (in $\%$) if we use the subsets $\rm SD_{i}$ instead of the subset $\rm SD_{6}$ as evaluation set. This way we can see the effect of scaling for the four different scale changes.

**Table 2:**
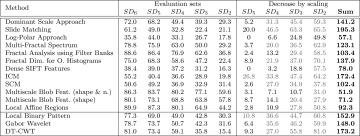
Results for the KTH-TIPS database

The column ‘Sum’ indicates the scale invariance of the methods

Gray letters indicate worst results

The decreases by scaling shown in Table 2 are relative to the results of using $\rm SD_{6}$ as evaluation set and are computed like in the previous subsection with the CUReT database:

$$\begin{aligned} d_{i} \, = \, 100 \, \frac{max((r_{\rm SD_{6}}-r_{\rm SD_{i}}),0)}{r_{\rm SD_{6}}},\qquad i\in \{2,\ldots ,5\}, \end{aligned}$$

where $d$ denotes the decrease by scaling and $r_{\rm SD_{i}}$ the result of using the subset $\rm SD_{i}$ as evaluation set. The last column ‘sum’ sums up the four decrease values when using $\rm SD_{i}$ with $i\in \{2,\ldots ,5\})$ instead of $\rm SD_{6}$ as evaluation set. In this way it is indicating how scale-invariant the methods are across different scale changes. The lower the sum, the higher the scale invariance of a method. The reason to replace negative decreases by scaling with zero ($\rm max(r_{\rm SD_{6}}-r_{\rm SD_{i}},0)$) is that we do not want that these negative decreases are lowering the sums. This could lead to wrong conclusions when comparing the sums of the methods.

The statistical significance is computed in the same way than at the CUReT database, but here we compare the outcomes of the images using $\rm SD_{6}$ as evaluation set to the outcomes of the images using $\rm SD_{i}$ with $\, i\in \{2,\ldots ,5 \}$ as evaluation set. If the outcomes of a method are significantly different, then the results (the decreases by scaling) are given in gray in Table 2.

We can see that for this experiment, most of the methods designed to be scale-invariant, are actually more or at least equally scale-invariant than the three scale-dependent methods (in contrast to the experiment with the CUReT database). Only the Slide Matching approach and ICM provide lower or at least similar low scale invariance than the three scale-dependent methods. It is hard to evaluate the scale invariance of the Log-Polar approach (as well as the Dense SIFT Features), since even using the same scale level ($\rm SD_{6}$) for the training and evaluation set provides poor results. Multiscale Blob features turned out to exhibit the highest scale invariance. Against all expectations, in this experiment the scale invariance turned out to be higher when using the scale-dependent number of blobs in addition to the scale-invariant shape as for using the scale-invariant shape alone. Also the Local Affine Regions provide reasonable scale invariance compared to the other methods in this experiment. Methods based on wavelet transform except for the Log-Polar approach (whose results are hard to interpret) are scale-dependent according to our results (methods designed to be scale-invariant as well as the scale-dependent ones). Once again, SCM is distinctly more scale-invariant than ICM. The three methods using fractal analysis provide average scale invariance compared to the other approaches.

Most methods are able to cope with small scale changes ($\rm SD_{6} \, \rightarrow \, \rm SD_{5}$ corresponds to a scale factor $f \, \sim \, 1.2$), for medium scale changes ($\rm SD_{6} \, \rightarrow \, \rm SD_{4}: f \, \sim \, 1.4$ and $\rm SD_{6} \, \rightarrow \, \rm SD_{3}: f \, \sim \, 1.7$, which is similar to the scale factor of the CUReT database) the results of most of the methods are significantly decreasing and for big scale changes ($\rm SD_{6} \, \rightarrow \, \rm SD_{2}: f \, \sim \, 2$) the results are even more decreasing. This coincides with the results of the significance tests. For most methods, there is no significant difference between the results without a scale change and the results with a small scale change and a significant difference between the results with no respectively medium scale changes. For all methods except the Log-Polar approach, whose results are hard to interpret, there is a significant difference between the results without a scale change and the results with a big scale change.

## Discussion

In this section we will discuss the reasons for the partly quite unexpected results. On the one hand we will analyze the methods and try to find the reasons why some of the methods are not as scale-invariant as they should theoretically be. On the other hand we will analyze the impact of the databases on the results.

### Analyzing the misclassifications caused by scaling

In this section we want to analyze which images are classified wrong because of scale changes. For this we analyze which images are classified correctly if the images of the training and evaluation set are gathered under same scale conditions, but are classified wrongly if the images of the training set are gathered under different scale conditions than those of the evaluation set.

In case of the CUReT database, it turned out that there are some frequently occurring types of misclassifications caused by scale changes. We define a type of misclassification as classifying images of texture class A into class A in case of identical scales in the training and evaluation set and as classifying images of class A into class B in case of different scales in the training and evaluation set. A and B denote two arbitrary, different texture classes.

**Fig. 11:**
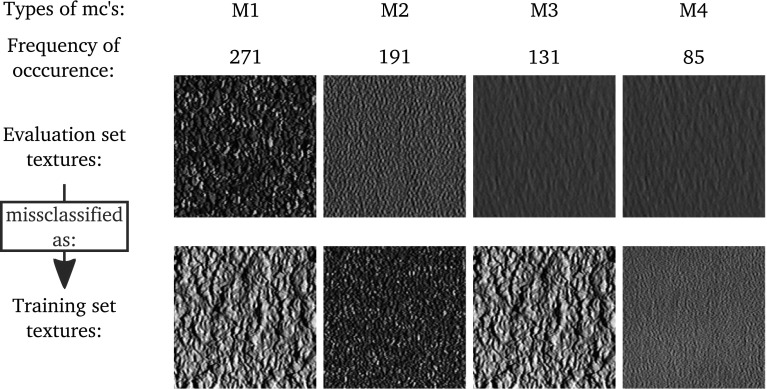
The most frequently occurring types of misclassifications (mc’s) caused by scale change

In Fig. 11 we see the four most frequently occurring types of misclassifications (M1–M4). All other types of misclassifications occur distinctly less often than these four. The “Frequency of occurrence” in Fig. 11 shows how often a type of misclassification occurs, summed up over all methods. So the highest possible number of misclassifications would be 1,380 (92 images per texture ×15 methods).

We can see in Fig. 11, that the sizes of local texture structures of images from materials of the (zoomed) evaluation set and of images from materials of the training set are quite similar in case of the four main types of misclassifications, especially for the types M1 and M2.

In Table 3 we can see the most fatal misclassifications of the methods caused by scaling (at least 30 % of the images of a texture class are classified into another texture class based on the output of a method). The numbers in brackets denote how often a type of misclassification occurs for the according method.

**Table 3:** The most fatal misclassifications (mc’s) of the methods caused by scale changes

Type of mc	Affected methods
M1	Dense sift features (71), slide matching (60),
	Fractal dim. for O. histograms (44) and ICM (41)
M2	LBP (35) and ICM (31)
M3	Fractal analysis using filter banks (45) and local affine regions (30)
M4	Multiscale blob feat. (shape and* n*.) (32)

Although there are several methods which are affected by the same type of a fatal misclassification (see Table 3), the methods’ misclassifications are different among our employed methods if we also consider the less fatal misclassifications among the four types M1–M4 and the remaining types of misclassifications apart from the four types M1–M4. So the misclassifications caused by scale changes are different among our employed methods.

In case of the KTH-TIPS database, we cannot limit the types of misclassifications to some dominating types of misclassifications, since there are too many different types of misclassification and none of them is occurring distinctly more often than others. The methods’ misclassifications differ among each other, even distinctly more as in case of the CUReT database.

### Analyzing the methods’ scale, viewpoint and illumination invariance

In this section we try to find and analyze the weak points of the different methods in general and especially with respect to their scale invariance. We already mentioned the obvious weak points of some methods with respect to scaling in Sect. 3. Now we want to find out the less obvious weaknesses of the methods by means of experiments.

The images of our two databases, the KTH-TIPS and the CUReT database, are not only gathered under different scale conditions, they are also gathered under different viewpoint and illumination conditions (different viewing directions and different illumination directions).

So the scale invariance of a method alone is not enough for a successful texture recognition, also viewpoint and illumination invariance is needed (and of course the general ability of the method to differentiate between images of different texture classes). In this section we will analyze the influence of scaling to the methods without any influence of viewpoint and illumination changes (Sect. 5.2.1), the influence of scaling and viewpoint changes without any illumination changes (Sect. 5.2.2), the influence of scaling and illumination changes without any viewpoint changes (Sect. 5.2.3) and the influence of combined scale, viewpoint and illumination changes (Sect. 5.2.4). In that way we are able to assess the influence of illumination and viewpoint changes to the scale invariance of the methods as well as the viewpoint and illumination invariance of the methods and their general ability for texture recognition. For our experiments we will use some novel image feature metrics especially designed to assess the scale, viewpoint and illumination invariance of the methods. Additionally we try to find the reasons for the differences of the results between the CUReT database and the KTH-TIPS database in Sect. 4 (Experimental analysis).

#### Analyzing the methods scale invariance

In the following experiment, we analyze the similarity of the methods’ feature vectors of texture images to the methods’ feature vectors of their scaled versions. In that way we are able to assess the influence of scaling to the methods, without any influence of other image transforms (in our case the variation of viewpoints and illumination conditions).

For our experiment we need a training set and a scaled version of this training set as evaluation set. So for each image of the training set there has to be one scaled version of itself in the evaluation set. The KTH-TIPS and the CUReT database fulfill this conditions. For each image of the training set there is one image of the evaluation set of the same texture class with same viewpoint and illumination conditions and a different scale level.

Our way to find out the influence of scaling of a method is to analyze the distances of the feature vectors of the texture images to the feature vectors of their scaled versions. Since the feature vectors of the different methods consist of different numbers of elements (features), since the elements of the feature vectors of different methods are differently high, and since the distances between the feature vectors are computed by means of different distance metrics, a direct comparison between the distances of different methods would be pointless.

First we compute the distances between the feature vector of an image from the training set and the feature vectors of the images of the evaluation set. Then these distances are ordered by size in ascending order, beginning with the smallest distance and ending with the highest. The distance between the feature vector of an image and the feature vector of its scaled version is in the midst of all the other distances and has a certain rank inside the ascending order of distances. We define the rank of an image as the rank of the distance between the feature vector of the image and the feature vector of the scaled version of the image. So e.g. rank $k \, = \,1$ means that the distance between the feature vector of the image (of the training set) and the feature vector of the scaled verson of itself in the evaluation set is smaller than the distances between the feature vector of the image and the feature vectors of the other images of the evaluation set.

As measure to evaluate the influence of scaling on a method, we introduce the ’rank of scale-similarity’ (RoSS), which is defined as the median of the ranks of the images of the training set (because of outliers we decided to use the median instead of the mean). RoSS is a measure for a method that shows the similarity between feature vectors of texture images and their scaled version. So the RoSS is not influenced by image transformations like e.g. viewpoint and illumination changes. The lower the RoSS of a method, the lower is the method influenced by scaling (and the higher is the scale invariance of the method).

**Fig. 12:**
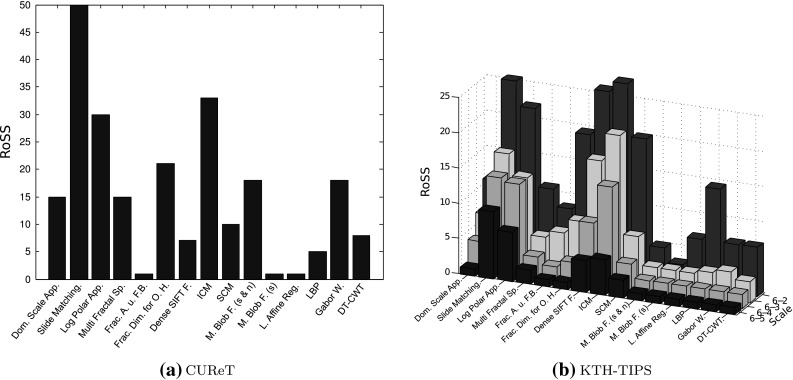
RoSS of the CUReT and KTH-TIPS database

In Fig. 12 we see the two diagrams showing the methods’ RoSS for the CUReT and the KTH-TIPS database. The training and evaluation sets are the same ones like used in our experiments in Sect. 4.2 (CUReT database) and Sect. 4.3 (KTH-Tips database). In case of Fig. 12b, the scale axis denotes the used training and evaluation set. E.g., Scale 6-3 means that we used scale level 6 ($\rm SD_{6}$ like defined in Sect. 4.3) as training set and scale level 3 ($\rm SD_{3}$) as evaluation set.

Of course, there is a different range of the RoSS values between the two employed databases, since the evaluation set of the CUReT database consists of 386 images (RoSS can be between 1 and 386) and the evaluation set of the KTH-TIPS database consists of 90 images (RoSS can be between 1 and 90). We are primarily interested in the RoSS values relative to each other and in the further discussion we always discuss the RoSS values relative to each other and not the absolute RoSS values when we compare the RoSS’s of the two databases.

As we can see in Fig. 12, the RoSS diagrams of the two databases are somehow similar. The similarities are higher if we only compare Scale 6–3 in Fig. 12(b) (KTH-TIPS) with Fig. 12(a) (CUReT). This is the most interesting case, since the scale difference between scale level 6 and 3 of the KTH-TIPS database is similar to the scale difference between the training and evaluation set of the CUReT database (scale factor $f \, \approx \,1.75$ in case of the CUReT database and scale factor $f \, \approx \, 1.7$ in case of the KTH-TIPS database).

The differences between the classification results of the experiments in Sect. 4.2 (CUReT database) and Sect. 4.3 (KTH-Tips database) are much higher than the differences between the RoSS results of the two databases.

Only the RoSS’s of the method Dense SIFT Features are highly different between the two databases (CUReT: low; KTH-TIPS: high). For both databases, the methods Slide Matching, Log-Polar Approach and ICM have high RoSS values, which leads to the assumption that these methods are not scale-invariant. The methods, Slide Matching and ICM, already turned out to be not scale-invariant in Sect. 4. In the experiment in Sect. 4.3 (KTH-TIPS database) we had problems to interpret the results of the methods, Log-Polar Approach and Dense SIFT Features, since their accuracies are already low if there is no scale difference between the training set and evaluation set (if the sets are identical). As we can see in Fig. 12, the Log-Polar Approach is definitely not scale-invariant (on both databases) and also the Dense SIFT Features are not scale-invariant (at least when used on the KTH-TIPS database).

The method Multiscale Blob Features using only the scale-invariant shape feature turned out to be least influenced by scaling for both databases, which corresponds to the results in Sect. 4. Also the methods Local Affine Regions and Fractal Analysis using Filter Banks are only slightly influenced by scaling. The three scale-variant methods (LBP, Gabor Wavelets and DT-CWT) provide average results for both databases. So, also this experiment shows that the scale invariance of most of the methods designed to be scale-invariant is not higher than the scale invariance of the methods which are not designed to be scale-invariant.

We can see from Fig. 12b, that most methods are able to recognize an image and its scaled versions as one and the same for small scale differences (Scale 6–5), but this ability is decreasing rapidly for increasing scale differences. In case of bigger scale differences (Scale 6–2 equates to a scale factor of 2) nearly all methods have problems to recognize an image and its scaled versions as one and the same. These results coincide with the results in Sect. 4.

#### Analyzing the methods viewpoint and scale invariance

In the following experiment, we analyze the similarity of the methods’ feature vectors of texture images to the methods’ feature vectors of their scaled as well as their original versions under different viewpoints. In that way we are able to assess the influence of scaling combined with the influence of varying viewpoints to the methods, without any influence of changing illumination conditions. By analyzing the similarity of the methods’ feature vectors of images to the methods’ feature vectors of their versions under same scale conditions but different viewpoints conditions, we are able to assess the methods viewpoint invariance.

As already mentioned before, our two databases consist of images gathered under different scale, viewpoint and illumination conditions. The images of the KTH-TIPS database are gathered under three different viewpoints and three different illumination conditions. Table 4 lists the 9 different viewpoint and illumination directions of the KTH-TIPS database per texture class and scale level.

**Table 4:** The nine images within each scale of a texture class in the KTH-TIPS database

Image number	Viewing direction			Illumination direction		
	Frontal	22.5° right	22.5° left	Frontal	45° from top	45° from side
1	x			x		
2	x				x	
3	x					x
4		x		x		
5		x			x	
6		x				x
7			x	x		
8			x		x	
9			x			x

Each image of the KTH-TIPS database has two other images (showing the same material) per scale level, gathered under same illumination and different viewpoint conditions and two images gathered under different illumination and same viewpoint conditions.

So, we can also test the influence of varying viewpoints and illuminations in a similar way as we tested the scale invariance by means of the RoSS measure. But this is only possible in case of the KTH-TIPS database. In case of the CUReT database, generally there are no image samples of a class gathered under same viewpoint or illumination conditions.

To test the influence of changing viewpoints (additionally to the influence of scaling), we introduce a new measure similar to the RoSS measure. This measure called “rank of scale-viewpoint-similarity” (RoSVS) will test the influence of varying scale and viewpoint conditions in a similar way as the RoSS measure tests the influence of scaling alone.

RoSS considers the distance between the feature vector of an image (of the training set) and the feature vector of its scaled version (in the evaluation set). Instead of that, RoSVS considers the two distances between the feature vector of an image and the feature vectors of the two images of the evaluation set showing the same texture class with different viewpoint and same illumination conditions. We define the (two) ranks of the image as the ranks of these two distances within the ascending order of distances between the feature vector of the image and the feature vectors of all the images of the evaluation set. The RoSVS measure is defined as the median of the ranks of all images from the training set.

If training and evaluation set are identical (Scale 6–6), then the RoSVS measures the influence of varying viewpoints alone, without any influence of scaling.

**Fig. 13:**
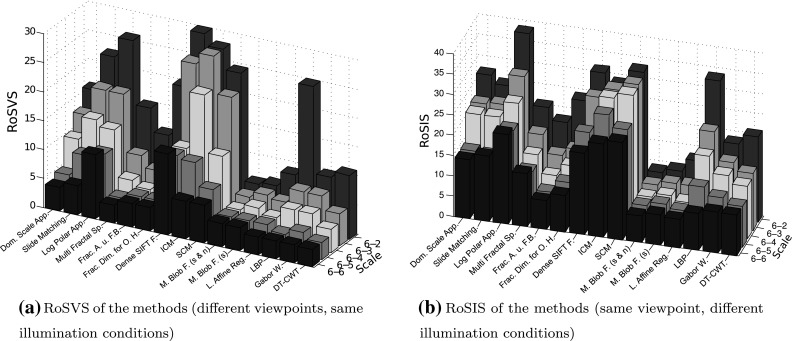
Two rank-based measures to test the influence of scaling and varying viewpoints (**a**) or to test the influence of scaling and varying illumination conditions (**b**) by means of the KTH-TIPS database

In Fig. 13a we see the RoSVS of the methods for the scale levels 2–6 of the evaluation set SD_2_–SD_6_ like defined in Sect. 4.3. As training set we always use SD_6_.

When we analyze the influence of varying viewpoints without any influence of scaling (scale 6–6 in Fig. 13a), we see that all methods except of the Dense SIFT Features and the Log-Polar Approach do not have problems with varying viewpoints.

When we compare Fig. 13a (RoSVS) with Fig. 12b (RoSS), we see that the values of the two measures are quite identical. The only real difference is that the RoSVS measures are all a little bit higher than the RoSS measures. This means that varying the viewpoints of images has only a minimal effect on the feature vectors of the methods. So varying viewpoints do not seem to be a problem for nearly all of the methods.

#### Analyzing the methods illumination and scale invariance

To test the influence of changing illumination conditions (additionally to the influence of scaling) to the methods’ feature vectors, we introduce a new measure similar to the RoSVS measure. By means of this measure we are able to assess the influence of scaling combined with the influence of varying illuminations and the influence of varying illuminations alone.

The measure to test the influence of varying illumination and scale, the “rank of scale-illumination-similarity” (RoSIS), is defined similarly to the RoSVS. The only difference is that RoSIS considers the two distances between the feature vector of an image from the training set and the feature vectors of the two images of the evaluation set showing the same texture class with identical viewpoint and different illumination conditions (RoSVS: different viewpoint and same illumination conditions). We define the (two) ranks of an image as the ranks of these two distances within the ascending order of distances between the feature vector of the image and the feature vectors of all the images of the evaluation set. The RoSIS measure is defined as the median of the ranks of all images from the training set.

If training and evaluation set are identical (scale 6–6), then the RoSIS measures the influence of varying illuminations alone, without any influence of scaling.

In Fig. 13b we see the RoSIS measures of the methods for different scale levels 2–6 of the evaluation set SD_2_–SD_6_ like defined in Sect. 4.3). As training set we always use $\rm SD_{6}$.

We can see in in Fig. 13b, that the methods are highly affected by varying illuminations. Actually, the effect of varying illuminations is nearly as high as the effect of scaling with scale factor $f \, = \, 2$ [scale 6–2 in Fig. 12b (RoSS)]. Averaging the RoSS values of the methods for scale 6–2 results in an only slightly higher value than averaging the RoSIS values of the methods for scale 6–6.

When we compare Fig. 13b (RoSIS) with Fig. 12b (RoSS), we see that the RoSIS’s of the methods are distinctly higher than the RoSS’s. We can also observe that the effect of varying illuminations is high for the methods that are highly affected by scale changes (Slide Matching, Log-Polar Approach, Dense SIFT Features, ICM and SCM) and low for the methods that are less affected by scale changes (Fractal Analysis using Filter Banks, the two variations of Multiscale Blob Features and Local Affine Regions). So varying illumination conditions seem to be a big problem for most of the methods, especially for those methods having also problems with scaling. For the two methods ICM and SCM we can observe (by comparing RoSS and RoSIS values) that the impact of scaling is smaller if the illumination conditions are changing as if the illumination conditions are constant. However, the reason for that could be the already huge impact of varying illumination conditions. Additionally, varying scale conditions only slightly decrease the already minor existing similarity of the feature vectors of same texture classes for the two neural network-based methods.

#### Analyzing the methods ability for texture recognition and their scale invariance

To test the combined influence of varying scaling, illumination and viewpoint conditions to the methods’ feature vectors, we introduce a new measure similar to the previous presented rank based measures. By means of this measure we are able to assess the methods’ ability for texture recognition, depending on how different the scale conditions are.

We proposed three rank-based measures (RoSS, RoSVS and RoSIS) to analyze the influences of scaling, varying viewpoints and varying illumination conditions. Additional to these three rank-based measures, we propose the “Rank of similarity” (RoS), a measure that compares the distances among the feature vectors of images of identical texture classes to the distances among feature vectors of images of different texture classes.

RoS considers the distances between the feature vector of an image of the training set and the feature vectors of images of the same texture class in the evaluation set with either different viewpoint conditions or different illumination conditions. In case of the KTH-TIPS database we consider 8 distances (9 images per texture class, the image with same viewpoint and illumination conditions is excluded) and in case of the CUReT database we consider 91 distances (92 images per texture class, the image with same viewpoint and illumination conditions is excluded). We define the ranks of an image (of the training set) as the ranks of these distances within the ascending order of distances between the feature vector of the image and the feature vectors of all the images of the evaluation set. The RoS measure is defined as the median of the ranks of the images of the training set. In case of the CUReT database, the RoS of an method can be between $\rm median(\{1,\ldots ,91\}) \, = \, 46$ (that means the 91 ranks of each image are the ranks from 1 till 91) and 322 (that means the 91 ranks of each image are the 91 highest possible ranks: $\{ 277,\ldots ,367\}$). In case of the KTH-TIPS database, the possible range of values of the RoS from a method is between 4.5 ($\rm median (\{1,\ldots ,8\}$)) and 85.5 ($\rm median (\{82,\ldots ,89\}$)).

**Fig. 14:**
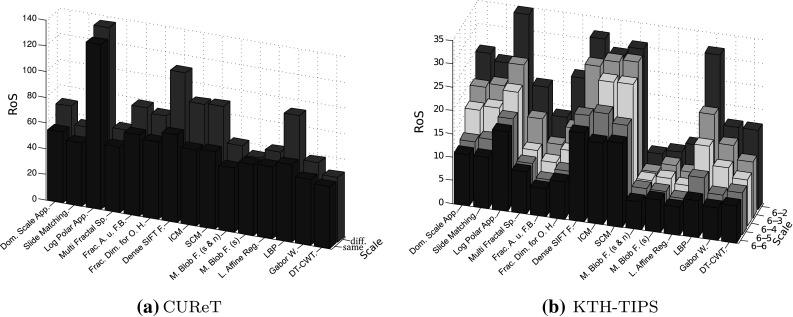
RoS as a measure to indicate the methods’ ability for texture recognition and the influence of scale changes

In Fig. 14 we see the RoS values for our two databases. The RoS of a method is a measure indicating its ability for texture recognition (like the accuracy in Sect. 4). The lower the RoS of a method, the higher its ability for texture recognition. In Fig. 14a, the two scales “diff.” and “same” mean that the training and evaluation sets are gathered under different or identical scale conditions, respectively (like setup 1 (same) and setup 2 (diff.) in Sect. 4.2).

We can see that the RoS results of the two databases for differently scaled training and evaluation sets are quite similar, the diagrams have the same peaks and look similar. In case of identical training and evaluation sets, the RoS results of the two databases are different. These differences could be caused by the fact that texture recognition is easier in case of the CUReT database, especially for identical training and evaluation set (the accuracies of all the methods except the Log-Polar Approach are almost at 100 % for the CUReT database in Sect. 4.2). In Fig. 14a (CUReT database) we see that the RoS values of the different methods are quite similar and quite low if the training and evaluation sets are identical (same scale), except for the Log-Polar approach, which is performing worse.

When we compare the RoS values for identical and different scales in Fig. 14a, we see that the RoS values for different scales are only slightly higher than those for same scales. Only the methods, Dense SIFT Features, ICM, SCM and LBP have distinctly higher RoS values if the training and evaluation sets are gathered under different scale conditions. So, the results indicate that only these methods are strongly affected by scale changes. In case of SCM this is a contradiction to the results in Sect. 4, where SCM was rated as a scale-invariant method in case of the CUReT database.

In general, the differences between the RoS values caused by scaling (the difference between the RoS values using identical or differently scaled training and evaluation sets), which are indicating the scale invariance of a method, are similar between the two databases. If there are differences between the two databases, then they are caused by the differences of the RoS results of the two databases using identical training and evaluation sets.

#### Comparing the RoS with the kNN-classifier

In this section we analyze the differences between the RoS measure and the results of the kNN-classifier. Additionally we try to find reasons for the differences of the results between the CUReT database and the KTH-TIPS database in Sect. 4.

As already mentioned before, the RoS of a method is a measure indicating its ability for texture recognition. A high RoS value indicates that the method performs poorly whereas a low RoS value indicates that the method performs well. So the RoS values shown in Fig. 14 are comparable to the classification accuracies of the methods shown in Table 1 (CUReT) and 2 (KTH-TIPS), with the difference that a high RoS value of a method indicates that the method performs poorly, whereas a high accuracy indicates that the method performs well.

Most likely, the kNN-classifier with the resulting accuracy is more suited to indicate a methods ability for texture recognition than the RoS. It is not essential that the methods’ feature vector of each image of a texture class is similar to the feature vectors of all the other images of the same texture class (necessary for good RoS results). It is enough if for each image there is a sufficient high number of images (depending on the application) from the same texture class with similar feature vectors (necessary for good kNN-classifier results).

So the main focus of RoS and especially its variations (RoSS, RoSVS and RoSIS) is not to find out the ability for texture recognition of a method (although it is also suited for it), it is to find out the weaknesses and the strengths of a method.

**Fig. 15:**
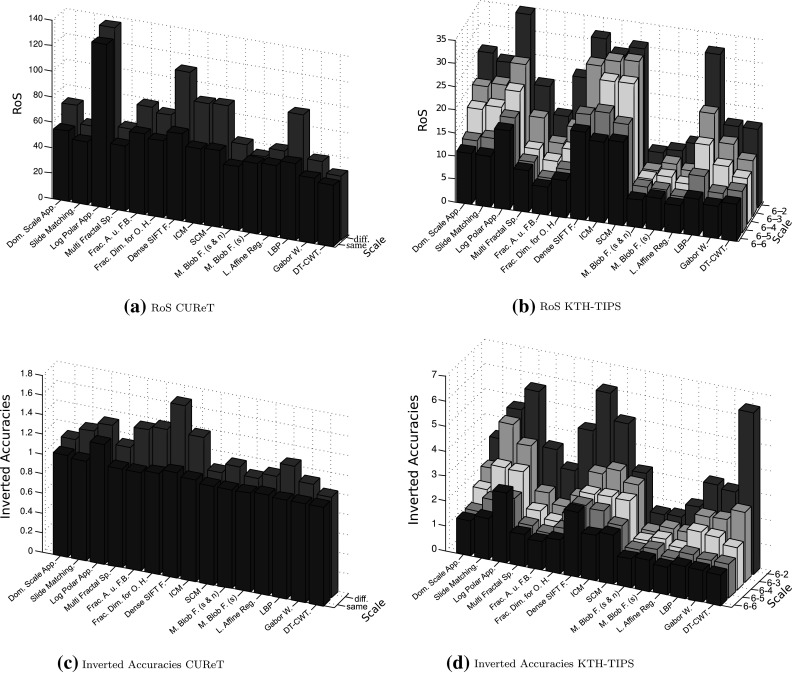
Comparing the RoS of the methods with the inverted accuracies of the methods

In Fig. 15 we compare the RoS of the methods with the inverted classification accuracies of the methods. The inverted accuracies (IA) are defined as follows: $\rm IA \, = \, 1/A$, where $A$ is the accuracy (e.g. $A \, = \, 50 \, \%: \, \rm IA \, = \, 1/0.5 \, = \, 2$). Low RoS values and high accuracies values indicate that an methods ability for texture recognition is high. So, a direct comparison between RoS and accuracy is only possible if one of the two measures is inverted.

We can see in Fig. 15 that the diagrams of the inverted accuracies and the RoS’s are quite similar. So both measures, accuracy and RoS, lead to similar conclusions with respect to the methods ability for texture recognition and their scale invariance. But with RoS and especially its variations (RoSS, RoSVS and RoSIS) we were able to find out some weaknesses and strengths (the amount of scale, viewpoint and illumination invariance of the different methods) of the methods.

As a matter of fact, there are only two real differences between the two measures. The kNN-classifier, which computes the accuracies of the methods, classifies an image either incorrect (0) or correct (1), whereas the RoS values of an image (the ranks of an image) are values between 1 and * N*−1 ($N$ is the number of image samples of a texture class), a type of grading where 1 is the top grade and * N*−1 the worst grade.

The other difference is that the kNN-classifier only considers the $k$ nearest neighbors, all the other images have no influence on the decision of the classifier, contrary to RoS. For example, let us assume that a methods resulting feature vectors of the images of a texture class with identical illumination conditions are quite similar, but the feature vectors of the images inside of a texture class which are gathered under different illumination conditions are quite different (as is actually the case for many of our applied methods). Then for low $k$ values of the kNN-classifier (low compared to the number of images in a class), the method is able to achieve high accuracies, since we only consider the k nearest neighbors of an image. Then the k nearest neighbors of an image woud be composed of the images of the same texture class with identical illumination conditions as the considered image. In case of the RoS, the results would be worse, since we consider the similarity between the feature vectors of all the images of a class (compared to the similarity of the feature vectors of images from different classes). This includes considering the similarity between feature vectors of images of a class with different illumination conditions, which is low in case of the method.

Roughly speaking, RoS considers the similarity of all the images of a class, whereas the kNN-classifier considers only the similarity between an image and its $k$ nearest neighbors. Especially for high $k$ values proportional to the number of images of a class, this can cause differences between the results of the two measures.

We can see in Fig. 15 that the RoS diagrams of the CUReT and KTH-TIPS database are more similar to each other than the accuracy diagrams. This means that the different number of image samples per texture class, and thereby the low $k$ value proportional to the number of image samples, could be one of the reasons for the differences between the results of the two databases in Sect. 4.

#### A comparison of the rank based measures

In this section we compare the previously discussed rank-based results. Additionally we try to find reasons for the differences of the results between the CUReT database and the KTH-TIPS database in Sect. 4.

Analyzing the rank-based results of the KTH-TIPS database, we see that the RoS results are quite similar to these of the RoSS and RoSVS results and especially similar to the RoSIS results. It’s interesting that the RoS values are slightly lower than the RoSIS values. In case of RoSIS we determine the similarity of the feature vector of an image to the two feature vectors of images from the same texture class with different illumination conditions, whereas in case of RoS we determine the similarity to the other feature vectors of the images from the same texture class with arbitrary viewpoint and illumination conditions. So, when a method has higher RoSIS values than RoS values, then this means that the effect of varying viewpoints is minimal to the feature vectors of the method as compared to the effect of varying illumination conditions.

Analyzing all the rank-based results of the CUReT database, we see that the values of RoSS are different to these of RoS for differently scaled training and evaluation sets (the RoSS values can be computed only for differently scaled training and evaluation sets). Firstly, the RoS values are distinctly higher and secondly the methods’ RoSS (relative to each other) are different to the methods’ RoS (also relative to each other). This indicates that in case of the CUReT database varying viewpoints and illumination conditions have a higher impact to the methods’ feature vectors as in case of the KTH-TIPS database. In fact there are more different illumination and viewpoint conditions in case of the CUReT database and the differences between the viewpoint and illumination conditions are distinctly higher as in case of the KTH-TIPS database. Since the RoS is a measure for the similarity of the feature vectors of a method inside a texture class, this includes the similarity among images with quite different viewpoint and illumination conditions. We already know from the KTH-TIPS database that the methods are much more affected by illumination changes as by viewpoint changes, so probably the differences between RoS and RoSS results are mainly caused by the quite different illumination conditions of the CUReT database.

#### The summation of the findings using rank-based measures

In this section we summarize all the findings using rank-based measures.

In Table 5 the methods’ scale, viewpoint and illumination invariance is rated based on the results of the rank-based measures RoS, RoSS, RoSVS and RoSIS. A ‘+’ stands for high invariance, a ‘$\circ$’ for medium invariance and a ‘−’ for low invariance. To rate the scale invariance, we used the results of RoSS and RoS using the CUReT and KTH-TIPS database. The viewpoint invariance is rated by means of the RoSVS measure and the illumination measure is rated by means of the RoSIS measure (both invariances are rated using only the KTH-TIPS database).

**Table 5:** The methods’ scale invariance, viewpoint invariance and illumination invariance

Method	Invariance		
	Scale	Viewpoint	Illumination
Dominant scale approach	$\circ$	$+$	$-$
Slide matching	$-$	$\circ$	$-$
Log-polar approach	$-$	$-$	$-$
Multi-fractal spectrum	$\circ$	$+$	$-$
Fractal analysis using filter banks	$\circ$	$+$	$\circ$
Fractal dim. for O. histograms	$-$	$+$	$\circ$
Dense SIFT features	$-$	$-$	$-$
ICM	$-$	$\circ$	$-$
SCM	$-$	$\circ$	$-$
Multiscale blob feat. (shape and* n*.)	$+$	$+$	$\circ$
Multiscale blob feat. (shape)	$+$	$+$	$\circ$
Local affine regions	$+$	$+$	$\circ$
Local binary pattern	$-$	$+$	$\circ$
Gabor wavelet	$\circ$	$+$	$\circ$
DT-CWT	$\circ$	$+$	$\circ$

As we can see in Table 5, most methods designed to be scale-invariant are not more scale-invariant than those methods that are not especially designed to be scale-invariant (LBP, Gabor Wavelet and DT-CWT). Most of the methods do not have problems with varying viewpoints, but big problems with varying illumination conditions.

We found three possible explanations for the different results of the two databases in Sect. 4:

1. The different number of image samples per class and scale level (Curet:92, KTH-TIPS:9).
2. Based on the accuracies of the kNN-classifier, texture recognition seems to be easier in case of the CUReT database (especially for identical training and evaluation sets).
3. The higher difference of the viewpoint and especially illumination conditions of the CUReT database, under which the images are captured.

### Analyzing the impact of the databases on the tested scale invariance

In this section we try to find the reasons for the different results with respect to the scale invariance of the two databases in Sect. 4. We already found three possible explanations for the different results of the two databases. In this section we look for additional, potential explanations for the different results of the two databases and verify which of the explanations are true and which can be abandoned.

**Fig. 16:**
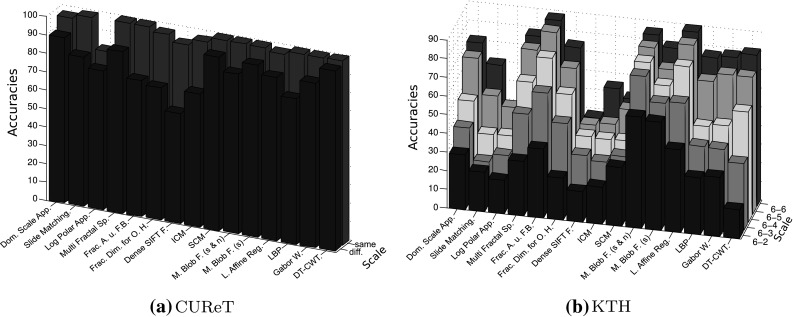
Results (Accuracies) of the performed experiments in Sect. 4 on the two databases

In Fig. 16 we show the accuracies using the kNN-classifier and in Table 6 we summarize the results of testing the scale invariance on the CUReT and KTH-TIPS databases in Sect. 4. Only the results indicating the scale invariance of the methods are shown in Table 6. The column ‘CUReT’ shows the results on the CUReT database and the column ‘KTH’ shows the results on the KTH-TIPS database. For the comparison of the results between the two databases, we only consider the results of the methods relative to each other. The best results in Table 6 are given in bold face numbers and the worst ones are in gray. Results that are hard to interpret are given in italic.

**Table 6:**
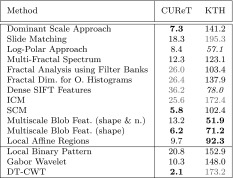
Results of the performed experiments on the two databases in Sect. 4, which are indicating the scale invariance of the methods

Bold letters indicate good results

Gray letters indicate worst results

Italic letters indicate results that are hard to interpret

When we compare the results of the two databases in Table 6, we see that the tested scale invariance of the methods is different for the two databases.

For some methods, the tested scale invariance differs a lot between the CUReT and the KTH-TIPS database (especially DT-CWT), but there are also methods which turned out to be clearly scale-invariant (the Multiscale Blob Features using only the shape of the blobs) or clearly not scale-invariant (especially ICM) for both databases.

In the following we analyze where these differences come from.

#### The different number of image samples per class

In this section we want to investigate if the different number of image samples per class is the reason for the different results between the two databases.

As already mentioned before, the number of image samples per class and scale level are quite different for our two databases. In the case of the CUReT database, there are 92 images gathered under different viewing and illumination conditions, and in the case of the KTH-TIPS database there are only 9 images gathered under different viewing and illumination conditions.

To verify if this is the reason for the different results, we construct a sub-database of the CUReT database, which consists of images of only 9 instead of originally 92 different viewing and illumination conditions per material. The results are computed in the same way as in Sect. 4.2, but this time we use the sub-database instead of the original CUReT database. The results are shown in the column “9 Conditions” of Table 7.

These results are (with respect to the ranking of the methods) similar to the original ones in Table 1 (92 different viewing and illumination conditions) and not closer to the results of the KTH-TIPS database. So, the assumption that the different number of images per texture class of the two databases causes the differences with respect to the tested scale invariance seems to be false.

#### The different extent of scale changes

In this section we want to investigate, if the different extent of scale changes is the reason for the different results between the two databases.

As already mentioned before, the scale difference between the original and scaled textures of the CUReT database is rather similar to the scale difference between the KTH-TIPS textures of scale level 6 (subset $SD_{6}$) and the textures of scale level 3 (subset $\rm SD_{3}$). So, if we compare the decreases caused by scaling for the same scale changes in the two databases, then eventually the differences of the results between the two databases decrease.

We can see that the decreases caused by scaling using $\rm SD_{3}$ instead of $\rm SD_{6}$ as evaluation set on the KTH-TIPS database (Table 2) are not more similar to the differences caused by scaling on the CUReT database (Table 1) than the decreases over all scale changes (column ‘Sum’ in Table 2), with respect to the ranking of the methods.

So, using the same scale changes for the two databases does not make the results more similar as compared to using different scale changes. As a consequence, the different scale changes are not the reason for the differences of the estimated scale invariances for the two databases. However, we already know from Sect. 5.2, that the rank-based results using identical scaled training and evaluation sets are quite different between the two databases, whereas the results using differently scaled training and evaluation sets are similar between the two databases, especially if $\rm SD_{3}$ is used as evaluation set on the KTH-TIPS database. As we can see in Fig. 16, also the accuracies using the kNN-classifier are more similar between the two databases if we compare the results for differently scaled training and evaluation sets as in case of identical scaled training and evaluation sets. So one of the main reasons for the differences of the results of the two databases could be that the accuracies of the methods for identical scaled training and evaluation sets are nearly all around 100 $\%$ in case of the CUReT database, contrary to the results of the KTH-TIPS database.

#### The different number of image classes

Another possibility is that the different number of materials (i.e. classes) could cause the observed differences. To test this assumption, we constructed three different subsets of the KTH-TIPS database, each consisting of four materials out of the 10 materials of the KTH-TIPS database.

**Fig. 17:**
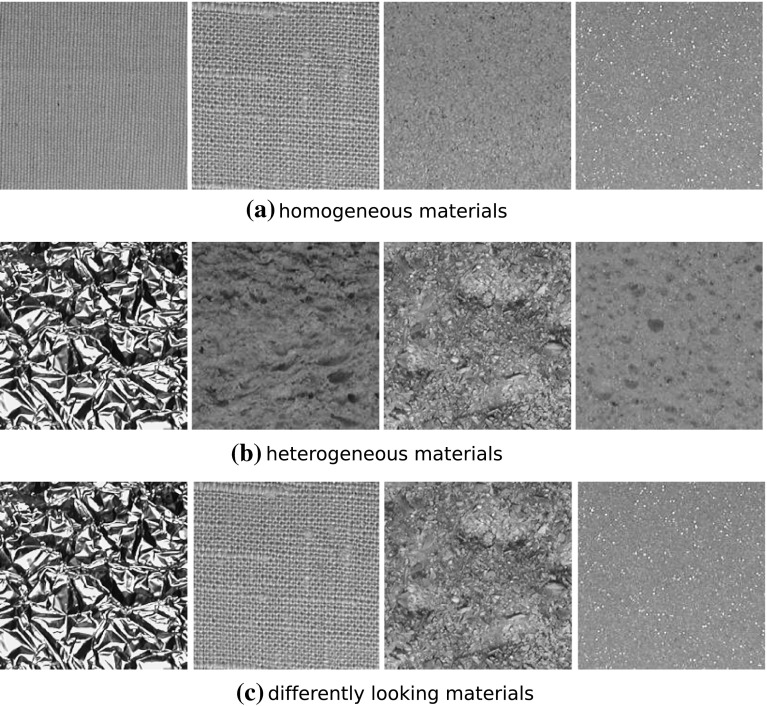
Subsets of the KTH-TIPS database consisting of 4 materials

The first subset consists of homogeneous materials. With homogeneous materials we denote textures that appear similar at each spatial location. The second subset consists of heterogeneous materials. With heterogeneous materials we denote textures that look differently at different spatial locations. The third subset consists of quite differently looking materials (see Fig. 17).

Tests were carried out in a similar fashion as for the original KTH-TIPS database. We only consider the summed up differences, which indicate how scale-invariant the methods are across different scale changes. The results for the three subsets and the average results ($\varnothing$) across the three subsets are shown in Table 7 labeled as “4 Materials”.

‘Hom’ denotes the subset with homogeneous materials, ‘Het’ the subset with heterogeneous materials, ’Diff’ the subset with different looking materials and ‘$\varnothing$’ denotes the average over the results of the three subsets. The best results are given in bold face numbers and the worst ones are in gray.

**Table 7:**
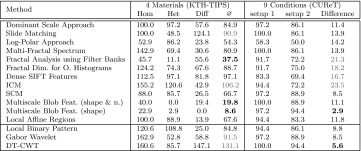
Results of testing the scale invariance for three different subsets of the KTH-TIPS database with only 4 materials per class (column “4 Materials”), and the results for only 9 different viewing and illumination conditions per material of the CUReT database (column “9 Conditions”)

Bold letters indicate good results

Gray letters indicate worst results

The average results in the column “4 Materials” in Table 7 are similar to those using 10 different materials (with respect to the ranking of the methods) and are not closer to the results using the CUReT database. The results of the three sub-databases are also not closer to the results using the CUReT database (with respect to the ranking of the methods). So, also the assumption that the different number of materials of the two databases causes the differences of the tested scale invariance is wrong.

#### The different image scales

Finally, we want to analyze if different image scales of the textures could be the reason for the different results in the two databases. The scale of the original textures of the CUReT database roughly corresponds to the scale of the textures with scale level 5 (subset $\rm SD_{5}$) of the KTH-TIPS database (as stated in the document describing the KTH-TIPS database^5^) and the scales of the scaled textures roughly corresponds to the scales of the textures with scale level 2 (subset $\rm SD_{2}$). We already carried out tests where we used $\rm SD_{6}$ as training set and computed the decrease by scaling using $\rm SD_{3}$ instead of $\rm SD_{6}$ as evaluation set (see Table 2). We already mentioned that these results are not closer to the results of the CUReT database than the results using the summed up decreases. The results also remain similar when we use $SD_{5}$ instead of $SD_{6}$ and $SD_{2}$ instead of $SD_{3}$. So, different scales are not the reason for the different results of the two databases.

#### Summing up the impacts of the databases on the tested scale invariance

Altogether, the different results for testing scale invariance of the two databases are not caused by different numbers of images, different scale changes, different numbers of materials or different image resolutions. As a consequence, the differences could be caused by different viewpoint conditions and especially by different illumination conditions, which are distinctly more different in case of the CUReT database. Unfortunately this assumption is hard to verify. But the differences could be also caused by the chosen materials or by the quality of the texture images (e.g. the amount of noise and blur they contain). For example, the images of the CUReT database contain much more noise than those of the KTH-TIPS database. The images of the KTH-TIPS database with lower scale levels (scale level 2 and especially scale level 1) are more blurred than those of higher scale levels. The degradation is caused by poor focusing [1]. In case of the CUReT database, there is no visual difference between the original and the scaled textures with respect to their blurriness.

One of the main reasons for the different results between the two databases is probably that the classification accuracies of the CUReT database for identical training and evaluation sets are all almost at 100 $\%$ except of the Log-Polar Approach, which is absolutely not the case for the KTH-TIPS database. As measured by the accuracies, texture recognition seems to be easier in case of the CUReT database, especially for identical training and evaluation sets. Also the RoS results are different between the two databases for identical training and evaluation set. So, the reason for the differences between the results of the two databases could be that texture recognition seems to be easier in case of the CUReT database, especially for identical training and evaluation sets.

## Conclusion

Based on the results of our experiments, the distinctly most scale-invariant method is ‘Multiscale Blob Features’ using only the shape of the blobs as feature. It is the only method that satisfies the expectations with respect to the scale invariance for both databases. All other methods provide worse results in case of the CUReT database, compared to those of the scale-dependent wavelet-based methods (Gabor Wavelet and DT-CWT). Especially the methods Slide Matching and ICM provide hardly any invariance.

When we compare the results of our two databases, we see that there are differences between the results of the two databases, especially with respect to the scale invariance of the methods. It would be helpful to conduct additional tests on other databases, to achieve a even more reliable assessment of the scale invariance of the methods and to verify the reasons for the differences of the results. The problem is that there are only two texture databases (the CUReT and the KTH-TIPS database), where textures are given at different scales and where the information about image scales is available. So, it is only possible to test scale invariance using these two databases.

It turned out that nearly all methods have big problems with varying illumination conditions, especially those methods that are less scale-invariant. Varying viewpoint conditions seem to be a smaller problem for the methods.

Overall, many methods that have been designed to focus on scale invariance turn out to fail in our experiments, since techniques which are not designed to be scale-invariant provide a similar extent of scale invariance. From this point of view, we have to state that techniques claimed to be scale-invariant should be actually tested for scale invariance in properly designed experiments, as suggested and conducted in this paper.

## Footnotes

1
http://rsbweb.nih.gov/ij/plugins/fraclac/FLHelp/BoxCounting.htm.

2
http://www.robots.ox.ac.uk/~vgg/research/affine.

3
http://www.robots.ox.ac.uk/~vgg/research/texclass/data/curetcol.zip.

4
http://www.nada.kth.se/cvap/databases/kth-tips.

5
http://www.nada.kth.se/cvap/databases/kth-tips/kth_tips.
